# Foreign

**Published:** 1841-06-26

**Authors:** 


### FOREIGN.

*Remarks on Hydrocele in the East, and the use of Tincture of Iodine as an injection.*—Hydrocele is so very prevalent a complaint amongst the Malays and natives of the Madras coast who reside in this island, but more especially among the latter, that those who are free from it may be said to form the exceptions. Both classes are so averse to apply for European assistance, that seldom an opportunity occurs of examining any hydrocele so small as the largest I have ever seen at home. It is found generally single, but not unfrequently double, and sometimes of such an enormous size as to extend more than half-way down the thigh. Great inconvenience is occasioned, from its weight and bulk, to progression; and on this account, as well as from the obstacle in all cases it forms to, and in some the total impossibility of, indulging the sexual appetite, they are induced to apply for relief. How it should be so common among these people I cannot explain, unless their frequent sexual indulgences;* and among the poorer classes, in whom the complaint is most prevalent, their entirely fish and rice diet, can have any influence in producing such complaints. It is met with at all ages, from puberty upwards, but much more frequently at middle life than at any other period. The very large hydroceles I have merely tapped, being afraid to inject any irritating fluid into so large a serous surface. On one occasion, from a single hydrocele, more than eight pints of serum were withdrawn. The smaller ones I have injected with the solution of sulphate of zinc (℥i to ℥xii;) wine and brandy, both in the pure and diluted state, and with the tincture of iodine (gr. xlviiii to ℥i;) but with none, to my knowledge, have I succeeded in making a permanent cure.† So little subject are natives to inflammatory action, that again and again, I have had to repeat the injection, before the necessary degree of irritation was produced; and frequently have injected the pure brandy without producing much. It is surprising, sometimes, to see violent irritations followed by the most trifling degrees of that action; and very unsightly wounds often heal kindly; but when inflammation does exist, they stand the depleting system very badly.

The tincture of iodine has not been, in my hands, more successful than the other kinds of injections, when used in the same way; but in these cases I have injected half a drachm, and allowed it to remain. The result has been, a speedy return of the fluid, with considerable pain; the last of which cases I again tapped four days afterwards, and the fluid that escaped was of a thicker consistence. I saw the patient two days ago, now twelve since the tapping, and there is no recurrence of fluid, but the testicle is considerably enlarged; the pain has, however, nearly subsided. My opinion of iodine is, that when used as other fluids are for injecting the tunica vaginalis, it will not be found a bit more effectual in producing a radical cure; and when allowed to remain and be absorbed, if a radical cure be thereby accomplished, we shall only be removing one disease to bring on another,—perhaps a more severe one—enlargement of the testicle.—*Dr. G. W. Smith, in Lancet.*

---

*Biphosphate of Magnesia and Ammonia in the Urine.*—At a meeting of the Medical Society of London, April 5, 1841, Dr. Alison related the following case:—A gentleman, æt. 30, of spare and delicate constitution, had been affected with dyspeptic symptoms for six years. During that time he had been under the care of several medical gentlemen, but without experiencing any permanent advantage. Dr. Roe treated him for several months with the usual tonics, but finding no benefit accrue from their use, recommended cold sea-bathing, which was rather hurtful than beneficial. On coming under the care of Dr. Alison, he complained of severe general debility, want of appetite, occasional uneasiness about the epigastrium, which he referred to flatus, and with which he was frequently affected. His food fermented, and became acid in the stomach; his countenance was pallid, and his frame altogether much emaciated; he complained of a sense of coldness at the back, and of dull uneasiness at the loins; his urine was high coloured and acid, and deposited a copious pink sediment; the urine voided more frequently than usual; tongue clean and moist; pulse weak, but natural in frequency; his habits regular and temperate. He was ordered to take, occasionally during

* It may be worthy of remark, that the Madras coast natives so frequently have recourse to indulgences of an unnatural kind, that these might perhaps, have been noted as inducing causes; but, on the other hand, the Chinese population here are equally vicious in their habits, yet are, as far as observed, comparatively exempt from this disease.—The loose dress of the natives might also have been considered a cause, were it not that the Madras coast people, from their infancy, use a kind of T-bandage to support those parts; whereas the Malays and Chinese, who do not, are, as already said, less subject to it than them.

† It may be noticed, generally, here, that it is, in a measure, impossible for the private practitioner to come to correct conclusions in the treatment of all classes of natives; the difficulty arising, first, from the great prejudice they have against seeking European assistance; second, the doubt which we always labor under that they will act up to the instructions given; and, lastly, their extreme negligence and ingratitude in not returning to inform us of the result.

the day, a mixture, consisting of compound infusion of gentian, carbonate of soda, and aromatic spirit of ammonia; and a pill, composed of Spanish soap, extract of henbane, and some aperient at bed-time. He was, likewise, ordered to take a teaspoonful of calcined magnesia any morning, when the pill of the previous night failed to produce a motion. Friction to the back with a coarse dry towel, moderate daily exercise in the open air, an animal diet, and half a glass of gin or brandy to be taken once or twice a-day, were also enjoined. In a few days he was much better, and had a good appetite; he did not make water so frequently, there was scarcely any deposit in his urine which did not affect litmus paper; his general health still improved. He called again in a few days: his general health was still improved; his urine was now light-coloured and clear, but when looked at in a phial, between the eye and the flame of a candle, presented a very remarkable and uncommon appearance. It was lustrous; there were hundreds of brilliant particles floating in the urine, which, on close inspection, were discovered to be very minute crystals, resembling benzoic acid in whiteness and lustre. When allowed to remain undistubed for a few minutes, these crystals sunk to the bottom of the phial, and formed a copious deposit, perfectly white, and about a line in depth. The urine was shown to Dr. Willis, who said that such a character of the secretion was rarely observed, and that he had seen it only two or three times. The crystals were those of the biphosphate of magnesia and ammonia. The aromatic spirit of ammonia and the calcined magnesia were discontinued; and, in the course of a few days, the urine had become quite healthy, affording no deposit, and not affecting litmus paper. The general health remains good, but the patient still complains of cold in the back and loins.

No discussion followed this case, except an inquiry made bv Dr. Bennett, as to whether too much attention was not paid to the state of the urine in cases of merely functional derangement of the digestive organs? He had found in some cases that an acid condition of the secretion was aggravated, and not removed, by the exhibition of alkalies, and *vice versâ.*

*Cases of sudden death occurring during Coition*, were related; one fell under the notice of Mr. Linnecar. In this instance the patient was a gentleman, about 38 years of age, suffering from phthisical symptoms; he was tall, and of spare habit of body. He had lately suffered much depression of spirits from some domestic calamity. On the night on which he died, he had walked home from the city with his wife, had eaten a light supper, and felt well. During the act of embracing his wife, he suddenly and instantly fell dead by her side. In this case the heart was healthy; there were tubercles in the lungs. Death had been produced by the rupture of the basilary artery. There was an immense quantity of blood at the base of the brain. The second case had been seen by

Mr. Hutchinson. It was that of a gentleman, about 60 years of age, of full plethoric habit; he had been in the habit of meeting a female and accompanying her home. On one occasion, during the act of coition, he suddenly fell dead. In this case, also, the basilary artery was found ruptured; the death was in both cases instantaneous.—*London Lancet.*

---

*Singular Case of Tumour in the Pelvis.* By Professor von D'Outrepont, of Würzburg.—A woman, 26 years old and well made, gave birth when 25 years of age to her first child without difficulty. Towards the end of her second pregnancy she again applied at the hospital in consequence of experiencing pain in the pelvic region. Vaginal examination discovered a hard and painful tumour, extending from the inner surface of the left ischium nearly to the corresponding point on the opposite side. It was hard, globular, even on its surface, and occupied the ascending ramus of the ischium and the descending remus of the pubis, and extended over the obturator foramen. It was impossible to reach the lower segment of the uterus, or to feel any part of the child.

The size and hardness of the tumour seemed to leave no chance of the birth of a living child, even by the induction of premature labour.—Professor D'Outrepont, who doubted whether the tumour was fibro-cartilaginous or a true bony exostosis, asked the opinion of many eminent men who saw the case. They did not express themselves with certainty as to its nature, and the patient refused to allow an experimental incision to be made into the tumour.

A short time before labour began, the tumour was thought to have become slightly compressible. When labour commenced, the professor called a consultation in which it was determined that unless a great change had taken place in the character of the tumour, an attempt should be made to remove it, or to cut away the bone if that should be found to be implicated, and as a last resource to perform the Cæsarean section.

On an examination being made, the right foot of the child was found to present, the cord was prolapsed, and did not pulsate. The tumour, however, was found to be so much softened that it was possible to pass three fingers through the outlet of the pelvis. Professor D'Outrepont brought down the foot, in doing which he found that the hips had compressed the tumor still more. The chief difficulty was experienced in extracting the head by means of the forceps, which gave the patient considerable pain. The child was still-born but was speedily recovered. After the birth of the child the tumour regained its former size, so that the placenta could not be expelled by the natural efforts, and it was necessary to introduce the hand in order to remove it.

The patient recovered rapidly, and returned ten weeks after her delivery, in order to have the tumour removed, which operation was performed by Professor Textor. The growth was found to be fibro-cartilaginous, and was connected neither with the bone nor the periosteum. It weighed 11½ ounces, and was so hard that none but they who were present at the patient's delivery, could have believed its previous softening possible. The patient was completely cured.—*Brit. and Foreign Med. Rev.*, *from Neue Zeitschrift fur Geburtskunde.*

*On the Action of the Oblique Muscles of the Eye.* By A. W. Volkmann.—[We have separated this portion forom the paper on the motor influences of the cerebral and cervical nerves, because it is not immediately connected with the subject there specially treated of, and that it may the more certainly attract the attention of the many who, since the operation for strabismus has been practised, have been engaged in experiments on the muscles of the eyeball. As far as we have seen, none of these observers have detected the mode of action ascribed to the oblique muscles by Professor Hueck and the author of this paper, and now, we believe, generally admitted in Germany. At the same time, none of the experiments lately performed are in any important degree opposed to the conclusions arrived at by these authors, and marked to all appearance with every necessary evidence of truth.]

In an admirable but little known treatise by Hueck, of Dorpat, (on the rotation of the eye on its axis, Dorpat, 1838,) it is shown that the office of the oblique muscles is to turn the eye upon its axis, so as to render it possible for the rays of light from any object to fall on identical spots on the retina, even when the head is inclined downwards; a condition on which, as is well known, singleness of vision depends. The observations (Nos. 2, 3, 4, 5,) related in the preceding paper, fully confirm this view. In the dog, calf, and other animals experimented on, the axis round which the eye rotates is the visual axis itself, or at least its position is so little different from that of the latter, that when the oblique muscles act alone no motion of the pupil is discernible. In man, a rotatory motion must also be produced by the action of the oblique; for since the muscles wind on the eyeball in the form of a semi-circle, rotation is unavoidable. But in man the axis of this rotation is not coincident with the axis of vision. While in the calf the direction in which the oblique muscles act intersects the visual axis at a right angle, in man the intersection is at an acute angle. The inferior oblique arising from the lacrymal bone goes outwards and backwards, comes in contact with the eyeball at a point directly beneath the axis of rotation, and is then prolonged backwards and outwards, and after describing a semi-circle, is attached to the outer and posterior part of the eyeball. The rotation which it produces will therefore have an axis, which may be considered as passing from the outermost part of the iris to the point of insertion of the optic nerve; in short, an axis which, instead of coinciding with the visual axis, cuts it, and passes across it from the front and externally backwards and inwards. The necessary consequence is, that when the inferior oblique acts *alone*, the pupil describes a portion of an arc of a circle; and, supposing that the view just advanced is correct, it must turn round the outermost point of the iris (which remains unmoved,) and be thus carried in a curved line upwards and outwards.

Albinus held this opinion respecting the action of the inferior oblique, but Bell, and with him many modern authors, have thought differently. The inferior oblique, they think, must move the eyeball so as to carry the pupil upwards and inwards, in the direction in which it is carried in wincing, and in which it is fixed during sleep. These motions, when automatic, are ascribed by Bell to the inferior oblique, and not to the combined actions of the superior and internal recti, which preside over voluntary motions exclusively. But the proofs for this opinion are insufficient. Bell divided the superior rectus in a rabbit, and found that the pupil was still moved upwards when the eye was irritated; but this might have been effected by the upper fibres of the retractor bulbi, (as in cap. 6.) He divided also the oblique muscles in a monkey, and it remained capable of all the voluntary motions of the eye, but the pupil on that side on which the inferior oblique was divided, was *scarcely distinctly* moved upwards in wincing. But a slight motion must require an organ for its performance not less than a great one does; and Bell has therefore unintentionally proved that the motion of the pupil in wincing may depend on the recti. But, in fact, this motion is never produced by the inferior oblique; for, 1st, that muscle could only move the pupil upwards and *outwards;* 2d, its action must be connected with the axis-rotation of the eye, of which no trace can be discerned in the act of wincing; 3d, the experiments already detailed prove that the contractions of the inferior oblique are not connected with the twitching upwards of the pupil; 4th, that same twitching upwards continues after division of the inferior oblique. In short, the position of the inferior oblique sufficiently indicates its action; it carries the pupil in the arc of a circle outwards and upwards.

In cases in which this rotation of the eye on its axis really takes place, it may be discerned in men as well as in animals, by a mode that Hueck pointed out. By fixing on a small vessel in the white of the eye, which (if possible) is directed transversely and horizontally across it, and then moving the head towards the shoulder, the vessel will be found not to alter its position; and it is therefore evident that the eye turns round in the direction opposite to that in which the head is inclined downwards.

The oblique muscles of the human eye, if they acted alone, would alter the position of the pupil, and consequently the visual axis; but they do not act alone. The straight muscles determine the direction of the visual axis, and this being once fixed, the action of the oblique muscles can be no other than that of turning the eyeball round its unchanging axis. —*Ibid, from Müller's Archiv.*, 1840. Heft iv. p. 480.

---

*Observations and Experiments on the Employment of Platina in Medicine.* By M. Ferd. Hoefer, M. D.—This is a lengthy but valuable contribution to medical therapeutics. The author first treats of platina in the metalic state, and of its principal compounds, and then examines its physiological action by experiments on animals, and on man in a state of health, and when suffering from disease. Several cases are given to illustrate the statements of the author; but our space prevents us giving more than his general conclusions, which are as follows:

1. The preparations of platina (chlorides) are poisons; the perchlorides in the dose of a scruple; the double chloride of platina and sodium in the dose of two scruples.

2. The chlorides of platina, (perchloride and double chloride of platina and sodium,) are less dangerous than the salts of gold, and the corrosive sublimate.

3. The perchloride of platinum in concentrated solution, produces acute itching on the skin, followed by a cutaneous eruption in the situation where the solution has been applied. Taken internally, it at first irritates the mucous membrane of the stomach, occasions cephalalgia, reacts on the nervous centres, and thus exercises a particular alterative action on the fluids of the economy.

4. The double chloride of platina and sodium does not produce local irritation on the skin. Taken internally, it does not react on the nervous centres in a manner so sensible as the simple perchloride. It more particularly augments the urinary secretion.

5. The perchloride of platinum is a remedy very efficacious in the treatment of syphilitic diseases; and particularly of those which are old, inveterate (constitutional.)

6. The double chloride of platina and sodium is well suited to the treatment of recent or primary syphilitic diseases. It is also very efficacious in the treatment of rheumatic affections.

7. Platina appears to rank in the class of medicines called alteratives, with gold, iodine, and arsenic. It differs from mercury in acting after a previous excitation, and as its administration does not entail any of the evils with which mercury is charged. The salts of gold, which appear to be poisonous in much smaller doses than the salts of platina, are, according to writers, only efficacious in certain cases of constitutional syphilis.

8. Platina, as an alterative medicine, is preferable to mercury and to gold.—*Ibid, from Gazette Médicale de Paris.*

---

*On the Employment of the Sulphate of Alumina in the Ulcerations and Inflammations of Mucous Membranes.* By M. Delmas.—The author of this paper concludes, from numerous observations, that alum dissipates the inflammation of mucous membranes; and that, applied to solutions of continuity of these membranes, it accelerates their cicatrization, while its action is never injurious when carefully applied. It also rapidly accelerates the cicatrization of cutaneous ulcerations, though in this case it is apt to induce pain and reaction. In syphilitic ulcers it is a most valuable topical application.—*Ibid, from Bulletin Géneral Theraputique.*

---

*Partial Injury of one of the Halves of the Spinal Marrow.* By M. Begin.—L., a robust old soldier, aged fifty-nine, received a wound from a sharp-pointed cutting instrument, in the back of the neck. He fell immediately, and being unable to raise himself, was obliged to be carried to an adjacent house. He said he had been knocked down by some heavy weapon, and that he had fallen, not in consequence of the wound in the neck, which he thought insignificant, but from the violent stunning which he felt at the instant he was struck. He fell *backwards* and on his right side; there was a slight bruise on the right elbow, but no other sign of external violence, although he attributed the impossibility of moving his right limbs to the shock and the bruises which he had received in his fall, or more still to the blows which he affirmed had been given him while on the ground.

The wound was united by plaster, and the patient, regarding his injury as a slight matter, refused to be bled. On the next day he was taken to the Val-de-Grace, where M. Begin first saw him, in the evening. He then said that he felt nothing but a little numbness of the right side. His pulse was slightly excited; the wound had healed, and he would not be bled. Cold was applied to the head and neck, and warmth to the feet, and antiphlogistic diet was ordered. He passed the night quietly. Next day, on examining the united wound more closely, it was found to be in a transverse direction, half an inch long, at the level of the fifth cervical vertebra, and nearly an inch from its spinous process. Its angles were nearly equally acute, rendering it probable that it had been made with a double-edged instrument; there was neither ecchymosis, nor swelling, nor heat, nor pain, nor hardness about it; the movements of the head and neck were perfectly free, and unaccompanied by pain, even when carried to a considerable extent. The patient felt a kind of weight in his right arm, and had creeping sensations in the hand; with a little difficulty, however, he could raise the arm, and move the fore-arm; but the fingers, half-flexed, could neither be opened nor closed completely. The right leg was absolutely motionless. A vague pain was felt along the right side of the chest, and there was perfect sensibility in every part of the body. The mind was clear: there was slight fever. The patient was bled freely both from the arm and locally; stimulants were applied to the feet, and cold to the head, and strict abstinence was observed.

On the third day after the accident, there was a slight power of moving the right great toe. But there was much more fever, and the patient was again freely bled. On the fourth day, after a very quiet night, he was a good deal better, but at night he became feverish, excited, and delirious. This condition was somewhat relieved by leeches; but next day he had a violent shivering-fit, followed by incomplete reaction; at night he had low delirium, with an unequal pulse, difficult, hurried respiration, and hiccup, and with these increasing, he died early in the morning of the sixth day after receiving the wound.

At the examination of the body, the wound externally was scarcely descernible, but more deeply its course became more and more evident by the effusion of blood in the adjacent tissues. In the middle of the right lamina of the sixth cervical vertebra there was a fragment of a knife, which projected backwards for about a line, and had its back directed towards the median line. Having removed all the cervical portion of the vertebral column, and cleaned its fore-port, the point of the same portion of knife was found projecting about one tenth of an inch between the sixth and seventh vertebræ. It had broken the upper edge of the body of the latter, and had stuck into, but not passed through, the posterior wall of the pharynx. The left side of the column was cut away, and the vertebral canal being thus opened, the medulla spinalis was found surrounded by pus, mixed with the spinal fluid, and its surface was softened above and below the wound. It had been struck by the back, or rather by the blunt edge of the blade of the knife, and the cut extended obliquely on the right side, from the level of the origin of the posterior roots of the nerves to the anterior median furrow. The posterior column was uninjured from the line of origin of the posterior roots to the posterior median furrow, while the anterior column was divided from the same point to the groove occupied by the anterior spinal artery.

The portion of the weapon that remained in the wound, was an inch and three quarters long, and three-fifths of an inch broad at its base: it was part of a short and very sharp dagger-knife.

The shock that must have accompanied the violent penetration of the arch of the sixth vertebra, and the intervertebral cartilage, as well as the part of the body of the seventh vertebra, perfectly explains the sensation of bruising and the stunning which the patient felt at the instant of receiving the wound.—Neither during life nor after death was there an erection of the penis.

[This case is interesting in every way, but especially in its obvious bearing on the functions of the columns of the spinal marrow, and the nerves respectively connected with them.] —*Ibid, from Bulletin de l'Académie de Médecine.*

---

*Cæsarean Operation saving the lives of both mother and child.* By Dr. Königsfeld, of Aachen.—At noon on the 28th March I was called to a woman in labour, who, in consequence of a remarkable narrowness of the pelvis, had had already, for several days, severe but ineffectual pains. I found that she was small, and bore various signs of having suffered from rickets. The pains were very severe; the woman could scarcely stand; the liquor amnii had not yet escaped. External examination discovered a remarkable bending inwards of the sacrum, and a furrow, nearly an inch and a half deep, formed by the projecting forwards of the two last lumbar vertebræ. The ossa pubis diverging downwards were so much pressed in upon the abdomen above, that all the walls of the abdomen above them hung over them like a sack. The vagina was hot and very narrow; the os uteri as large as a dollar, and very hard; the membranes were moderately tense, and the child's head could be obscurely felt behind them, in the same position as, according to the midwife, it had occupied for the last four days. An accurate measurement showed that the conjugate diameter of the pelvis was only between two and two inches and a half long, and I therefore determined on the Cæsarean operation as the only means of effecting delivery; for, even if perforation and embryotomy could have been performed, they were forbidden by the child being still alive, and the patient rapidly sinking.

The incision was commenced half an inch on one side of the umbilicus, was then turned to the linea alba, and, extending for six inches, terminated close to the os pubis. The uterus presented at the wound, and was divided in the same direction as the external incision, and opened for an inch at its upper part. The membranes were now broken somewhat lower down, carrying the finger along on the knife, and the waters were allowed to flow *per vaginam;* the wound in the uterus was then carried on, and the membranes separated lower down. The right shoulder of the child now pressed into the wound, and was slowly pushed on, bringing after it the right arm, the chest, the buttocks, and the lower extremities; the head stuck in the wound, and required great care in removing it. The intestines, which were forcibly pushed forwards, were held back by large sponges, and the placenta was removed with the hand as soon as possible. The hæmorrhage throughout the operation was inconsiderable; and after as much as possible of that which had flowed had been pressed out of the abdomen, the wound was united by suture and plaster. The operation lasted twelve minutes. Four hours after, bloodletting was found necessary to calm the violent inflammatory excitement that had commenced; but after this, with only mild treatment and perfect abstinence, (for hunger, says the author, is the best antiphlogistic,) the case went on perfectly well; the wound was healed, and the patient entirely recovered four weeks after the operation. The child also was healthy from its birth, and grew very rapidly.—*Ib., from Casper's Wochenscrift. Dec.* 5, 1840.

---

*On Kyanised Timber.* By Dr. SCHWEIG, of Carlsruhe.—The discovery made in England by Mr. Kyan, namely, that wood might be preserved from dry-rot and the destructive attacks of insects, has now, we are told by the author, come into such general use on the continent, as to require an examination into its influence on public health. Kyan's process consists in saturating wood thoroughly, by long immersion, with a solution of corrosive sublimate, in the proportion of two pounds of sublimate to one hundred measures of water.

In regard to the influence of this process on public health, the following points require attention:

1st. From the ready absorption of this poisonous liquid through the skin, the mere manipulation of the timber exposes the workmen to much danger.

They should be warned not to dip their hands in the liquid more than can be avoided. They should wear linen, which ought to be frequently washed; the water used in washing either their persons or their linen should have muriate of ammonia dissolved in it, so as to precipitate and separate the poison more effectually. Lastly, the earth on which any of the poisonous liquid may fall should after a time be dug up and buried in a deep hole.

2d. As a matter of medical police, it is proper to enquire whether under certain circumstances, mercury may not be volatilized from wood thus prepared, and produce its usual dangerous effects upon those exposed to the vapour.

The occurrence of prejudicial effects from this cause has been denied, because the crews of vessels built of Kyanised timber have returned healthy after long voyages, even in tropical latitudes. Where there is a free access of air, as in the wood used in railways (sleepers,) no danger from this source is to be apprehended; but when the wood is employed in the construction of close apartments, in damp or ill-ventillated places, the case is different. Experience may not have hitherto shown that dangerous consequences have followed; but if there be reason to suspect the possible occurrence of these on physical grounds, this is sufficient to justify a government in interfering to prevent the use of such timber for the construction of houses.

3d. It need hardly be said that wood thus impregnated with corrosive sublimate, is wholly unfitted for the making of vessels to hold articles of food or drink, either for the use of man or animals.

4th. A fourth and very important consideration is, that Kyanised wood when used up cannot be safely employed for fuel like ordinary wood; the mercury contained in old wood might be thus volatilized and spread in vapour through the apartments of a house, leading to the slow destruction of life by poison, by giving rise to protracted illness. It could only be safely burnt in close stoves or vessels, where there was a free current of air to carry off the mercurial vapour.

The chips or cuttings of wood of this description, might unknowingly be employed as fuel by labourers and others engaged in working the timber.

From the preceding remarks it may be inferred, that some precautions should be taken by a government in allowing the application of this process; and it is at the same time a duty to ascertain whether some harmless substitute equally preservative may not be found for the deadly drug at present used.

The author says that a substitute of this kind is now well known and much employed, namely, the sulphate of copper. This effects the same chemical changes on the soluble principles of wood as corrosive sublimate; and although being a poison the wood cannot be used for the manufacture of domestic utensils, yet the metal copper is fixed, and when the wood after use is burnt, there is no fear of its giving out poisonous vapours. Owing to this it might also be safely employed in the construction of houses or in buildings for the use of man and animals. It is then a fair question whether a government ought not to prohibit the use of timber thus saturated with sublimate, and recommending as a substitute a solution of the sulphate of copper.

[We consider this to be a very important paper, and deserving the attention of a board of medical police, if there were such in this country. Fortunately, the process in England is patented, and this circumstance has, to the great benefit of the community, restricted its application. Should the use of Kyanised timber spread after the expiry of the patent, which we believe there is little fear of, as the process is likely to be superseded by a more effective one, we may expect that cases of poisoning will be frequent, not so much in consequence of the fragments of wood being used for fuel, as from its being ignorantly used in the construction of barrels, tubs, cisterns, spouts for the holding of water, milk, wine, or other liquids. It is absurd to say that its use should be restricted to out-of-door purposes, since you cannot provide knowledge for the lower classes of artificers; and, whether by accident or through ignorance, Kyanised wood will be frequently used for purposes not contemplated by the inventor of the process. The bare possibility of a danger of this sort occurring, is, as Dr. Schweig observes, at once a sufficient justification for a government to interfere.

We believe that the author is mistaken in thinking that sulphate of copper is equally effective as corrosive sublimate, imperfect as even this is, and he seems quite ignorant of the more recent discovery of Sir William Burnett, that *chloride of zinc* is greatly superior to both in the preservation of wood, and yet more in the preservation of sail-cloth, and cordage. The great superiority of this agent is yet further enhanced by its want of poisonous qualities. We believe Sir William Burnett has obtained a patent for this discovery, and that it is deservedly taking the place in this country of the more dangerous process of Mr. Kyan.] —*Ibid. from Henke's Zeitschrift, f. d. S. A.*; 1, 1840.

---

*On the Removal of Foreign Bodies in the Joints by means of Subcutaneous Incisions.* By Dr. Goyrand, of Aix.—This new method of operating was suggested by the fact, that incisions made into the joints through such small external openings that the air cannot obtain admittance to the cavity, are never productive of that severity of inflammation which follows wounds with wide external apertures, and which, in the operations hitherto performed for this disease, has so often terminated fatally. The proceeding recommended by the author consists in pushing the loose body (if in the knee-joint,) into the synovial pouch above, and to the outer side of the patella, beneath the vastus externus muscle, and while an assistant holds it fixed there, passing a narrow knife through the skin at some distance above the joint, and through all the intermediate tissues down to the foreign body. Without enlarging the opening in the skin, the synovial membrane and adjacent tissues over the loose substance are now to be freely divided, till, by the pressure on the latter, it slips out of the joint through the wound, and lodges itself in the subcutaneous cellular tissues, or in some of the other tissues between the skin and the joint. After this the patient must remain at rest for several days, (the small external aperture being merely covered by sticking-plaster,) till all chance of inflammation occurring has passed away. The foreign body dislodged from the interior of the joint, will form a cyst for itself, and remain in its new position without producing any annoyance; but, if it should be deemed necessary, it may easily be removed by a single incision through the skin over it, which will no longer be likely to excite any inflammation of the joint itself.

The author details a case in which this method of operating was adopted with perfect success. Two loose cartilages were dislodged, and pushed into the tissues adjacent to the joint. One was subsequently removed by an incision through the skin; the other was allowed to remain, and it produced no inconvenience whatever. Through the whole course of the treatment the patient had not a bad symptom, although less precautions were adopted than are usual in this description of cases.

[We understand that Mr. Syme, of Edinburgh, performed this operation, and with complete success, without previous knowledge of M. Goyrand's proceeding.]—*Ibid, from Annales de la Chirurgie Francaise et Etrangère.*

---

*On the Efficacy of Chloride of Zinc in the Treatment of Acute and Chronic Gonorrhœa.*—By M. Gaudriot.—After a large number of observations, the author of this memoir concludes that the chloride of zinc, properly diluted, has a remarkable power in curing simple gonorrhœa of the urethra and vagina, also in dilating the urethra, and thereby ameliorating strictures. He believes that it is always by an inflammation *sui generis* that the discharge is cured, and that injections of the chloride of zinc determine a series of phenomena not produced by other caustics. It is only necessary to use a few drops of injection, as the extremity of the urethra is the only part to which it should be applied. To combat vaginal gonorrhœa in women, M. Gaudriot proposes a new proceeding, which consists in the use of solid vaginal suppositories, composed of a paste made to melt easily, and containing a certain quantity of liquid chloride of zinc and sulphate of morphine.

In men the author employs the following formula:

Liquid chloride of zinc, 24 to 36 drops.
Distilled water, four ounces.
Agitate and filter through paper.

A small quantity of this solution should be injected about an inch along the urethra, two or three times a day.

The vaginal suppository which he employs is formed of

Liquid chloride of zinc, 5 drops.
Sulphate of morphine, half a grain.

Mix with three drachms of the following paste:

| | |
|---|---|
| Mucilage of gum tragacanth, | 6 parts. |
| Powdered sugar, | 3 " |
| Starch powder, | 9 " |

To cure radically a gonorrhœa in man, it will ordinarily suffice to apply two injections a day for two or three days. The first injections are almost always followed by more or less swelling of the glans penis, but this does not prevent their continuance.

In women, four or six suppositories, one being introduced every day, or every second day, suffice to obtain a cure. The first introductions frequently occasion a swelling, with more or less heat of the vulva, but these phenomena are soon completely dissipated. Emollient baths have in most of the cases been employed for this purpose.—*Ibid, from Journal des Connaissances Médicales Pratiquesetde Pharmacologie.*

*Mode of Action of Cubebs and Copaiba.* By M. RICORD.—At the sitting of the Royal Academy of Medicine, on the 8th of September last, M. Ricord showed a design of a case of accidental hypospadias, resulting from a urinary abcess. The patient affected with this infirmity having contracted a gonorrhœa, it gave rise to some curious observations. The discharge first showed itself in the vesical portion of the urethra, afterwards the part situated before the solution of continuity was invaded in its turn. Treated by copaiba, the vesical portion was soon cured, but the disease continued in the other part, and afterwards communicated it to the portion already cured.—Cubebs was administered, and the discharge again ceased in the posterior portion of the canal. These facts show, according to M. Ricord, that cubebs and copaiba cure syphilitic discharges by the principles or properties which they communicate to the urine, and of which the urethra receives the influence by the passage of that fluid.—*Ibid, from Arch. Gen. de Méd.*

*On the Origin and Mode of Development of Zoospermata.* By M. LALLEMAND.—The zoospermata secreted by the testicles are susceptible, as all the products of secretion, of modification by all severe diseases, and by any cause greatly or protractedly disturbing the economy. Thus they diminish in number, in volume, in density, in vitality, according to the severity of the affection, and resist, more or less, putrid decomposition. They may become very scarce, and even be replaced by pyriform, ovoid, or spherical bodies. They are very mobile in their living state, and remarkable after death by their brilliant aspect, and the regularity of their forms. At the age of puberty these globules precede the appearance of the complete zoospermata, and replace them in old age and in certain pathological states, by which it appears that they are incomplete unformed secretions. The same phenomena are observed in healthy animals at each periodical return of heat. The zoospermata are most numerous in the secretory canals of the testicles, where they are almost dry, and heaped one upon another, most frequently in groups, of which all the heads are directed towards the epididymis, and the tails towards the surface of the testicle. In the vasa deferentia they separate and become more mobile and perfect. The fluids furnished by this canal, by the vesiculæ seminales, the prostate, and Cowper's glands have no other function than to favour their dilution and movements. The most perfect are always found the nearest to the excretory orifice. The most mobile, those which live the longest, are those which are voided during copulation. The ovules, secreted by the ovaries, are also, according to M. Lallemand, living bodies before fecundation; they attain perfection in the oviducts after their separation from the ovary, and comport themseves exactly as the zoospermata.

[From the last proposition the editor of l'Expérience dissents, stating that the zoospermata are complete beings, endowed with a peculiar independent life: but the ovules, on the contrary, are fragments of the ovary, which only participate in the general life of the individual, as all other parts of the organism, and do not acquire the independent life of spermatic animacules until after fecundation.]—*Ib., from L'Expéreence.*

*Radical Cure of Varicocele by means of Invagination and Shortening of the Scrotum.* By Dr. LEHMANN.—The mode of operating here recommended is very similar to that which the author has in several cases adopted successfully for the radical cure of hernia. A portion of the relaxed and elongated scrotum is to be pushed up on the fore-finger of the left hand, and *invaginated* into the part above it, till the finger reaches the external abdominal ring. Holding the parts in this position, a broad curved needle, with a double thread passed through an eye near its point, is to be carried along the left finger, through the bottom of the inverted portion of the scrotum, and to be made to penetrate it and the integuments immediately over the external ring. The thread is then to be removed from the eye, and the needle drawn back and again carried through the inverted portion of the scrotum and the integuments, at a distance of about half an inch from the parts previously penetrated. The threads passed through the two apertures being now drawn, the invaginated portion of scrotum may be pulled up to any desired height; in general it must be drawn very nearly to the external ring; for in the subsequent progress of the cure, the relaxation of the parts is so great that the folds and wrinkles thus produced are almost entirely obliterated. The threads then having been drawn must be knotted, and the parts thus tied up must be left for eight or nine days, under a soothing and cooling regimen, by which time adhesion will have taken place between them and the cut edges, and, by the excoriation which the discharge produces between the opposed surfaces of the inverted portion of the scrotum and that into which it is pushed.

The author relates six cases in which his mode of operating was adopted with success, but in none of them had sufficient time elapsed before the publication of the paper, to enable one to say whether this measure was likely to be more permanently beneficial than the others which have been proposed for curing varicocele by bringing on the same change in the scrotum. Of all these the simplest, and, as far as we have seen, the most successful, is that recommended by Mr. Wormald. (See Brit. and For. Med. Rev., vol. vi. p. 274.—*Ibid, from Med. Zeitung.*

---

*Practical Considerations on the Treatment of White Swelling.* By Dr. Forget.—The only novelty in this paper is an account of the employment of the muriate of baryta (chloride of barium,) in cases of chronic arthritic disease, as used in the hospital of La Pitié. The author considers it to be a most useful medicine, both in the acute and chronic state of white swellings, especially in scrofulous patients, and believes that in some cases it has alone sufficed to effect a cure, while usually great amendment has followed its use, though the cure may not have been complete. The usual dose has been six decigrammes, continued for a month. The circulation is very generally lowered, the pulse being depressed from sixty or eighty to forty, or even twenty-five [?] in the minute. Local bleeding and compression have been advantageously combined with the employment of the baryta.—*Ibid, from Bulletin Général de Thérapeutique.*

---

*On the Structure of the Placenta.* By Dr. John Reid, F. R. C. P. D., Lecturer on Physiology, &c.—It is with great pleasure that we find ourselves again called upon to notice the results of Dr. Reid's industry and skill in the prosecution of anatomico-physiological inquiries; and our pleasure is the greater in the present instance, because his recent labours have given what is to our minds a very satisfactory solution of a long-disputed question of no slight interest, the connexion between the fœtus and the uterus of the human female. We shall briefly state the facts observed by Dr. Reid, and his inferences from them.

In dissecting the uterus, with the attached placenta, of a woman who had died suddenly during the seventh month of pregnancy, Dr. Reid's attention was attracted towards a number of rounded bands, passing between the uterine surface of the placenta, and the inner surface the uterus. These bands [which were distinct from the utero-placental vessels described by the Hunters] were generally observed to become elongated, thinner, and of a cellular appearance when put on the stretch, and were easily torn across; whilst at other times, though much more rarely, *they could be drawn out in the form of tufts from the mouths of the uterine sinuses.* Some of these tufts were found to be much more elongated than others, occasionally extending an inch from the open mouth of the sinus by which they had entered, and even passing into a neighbouring sinus, and sometimes scarcely dipping into the sinus. These tufts were clearly ascertained to belong to the fœtal system, being filled by injection of the umbilical veins and arteries, bound together so as to form apparently but one set of filaments,—a structure exactly corresponding, therefore, (as Dr. Reid justly remarks,) with the branchial filaments of aquatic animals. These tufts lay within the uterine sinuses in some degree loose, but were observed to be bound down at various points by reflexions of the inner coat of the venous system of the mother upon their outer surface (just as, it may be pointed out, the intestines are supported in the abdominal cavity, by the reflexions of the peritoneum.) It is on account of this kind of union, that the tufts cannot readily be withdrawn from the sinuses, the connecting vessel usually giving way in preference.

With this clue Dr. Reid proceeded to examine minutely the structure of the placenta itself; and he succeeded in demonstrating that the greater part of its substance exactly corresponds with the tufts just described; being composed of bundles of filaments interwoven with each other, each filament being composed, like those of the tufts, of a ramification of the umbilical artery and vein. There is no cellular or any other tissue filling up the spaces between the branches of the fœtal placental vessels; the so-called cells being merely the interstices between the interlacing tufts, which tufts do not seem, in spite of their close approximation, to be actually connected with each other. Upon tracing the coats of some of the utero-placental veins, which contained none of the tufts, and of the curling arteries, through the decidua it was found that they were reflected over these prolongations of the umbilical vessels within the placenta; just as the lining of the uterine sinuses is reflected over the tufts which project into them. The interior of the placenta is thus shown to consist of the ramications of the umbilical vessels, the blunt terminations of which are ensheathed in prolongations of the inner coat of the vascular system of the mother, or at least in a membrane continuous with it.

If we adopt this view of the structure of the placenta, the blood of the mother when it passes into that organ, through the curling arteries of the uterus, may be regarded as entering a large sac formed by the inner coat of the vascular system of the mother, and this is intersected in many thousands of different directions, by the placental tufts prosecting into it like fringes, and pushing its thin wall before them in the form of sheaths, which thus closely envelope both the trunk and each individual branch composing these tufts. Round this sac the maternal blood is returned by the utero-placental veins, without having been extravasated, or without having left her own system of vessels. Into its cavity, the tufts of the placenta hang, like the branchial vessels of aquatic animals, and are bathed in the blood of the mother, just as are those tufts which project into the sinuses. This sac is protected and strengthened on the fœtal surface of the placenta by the chorion; on the uterine surface of the decidua vera (of the existence of which in this position Dr. Reid has satisfied himself,) and on the edges or margin by the decidua reflexa. The blood of the mother and that of the fœtus can readily act on one another through the spongy and cellular walls of the placental vessels and the thin sac ensheathing them ; just as the blood in the branchial vessels of aquatic animals is acted on by the water in which they float. According to this view of the structure of the placenta (which, when once understood, is found to be characterized by extreme simplicity, as well as by conformity to analogy,) there is no proper division into the fœtal and maternal portions, since tufts of placental vessels with their blunt terminations may be found immediately under the chorion covering its fœtal surface, as well as towards the uterine surface.

From a small number of observations made upon uteri and placentæ of different ages, Dr. Reid is disposed to conclude (though he by no means wishes to state it as a positive fact) that the degree in which the tufts of fœtal vessels project into the uterine sinuses increases with the advance of gestation. When the placenta is detached, at the conclusion of delivery, it would seem that most if not all of the tufts are retained within the sinuses, being kept there by the connexions already mentioned. We think it not unlikely, however, that some may be occasionally drawn out, but escape observation; and we would suggest to those of our readers whose practice affords them facilities for so doing, that they take pains to ascertain this fact, by immersing the placenta in the water, as soon as may be after its separation, and carefully observing whether any such tufts hang down from the placental surface, when it is placed horizontally and on looking downwards. Whether this be the case or not, however, the existence of the tufts has been placed beyond a doubt by Dr. Reid, who has satisfied Professors Alison, Allen Thompson, and J. Y. Simpson of their character; and Dr. Sharpey has given the weight of his testimony, based upon independent observation. Feeling, as we do, great confidence in Dr. Reid's caution and sagacity as well as anatomical skill, we have little hesitation in according with the general view of the structure of the placenta, by which he has been led by this interesting discovery; and we trust that it will receive full confirmation from the researches of others.

Dr. Reid's paper contains, besides an account of his own researches, a summary of opinions of others on this *questio vexata*. Of these, Weber's views appear to present the nearest approach to his own. Weber had not noticed any prolongation of the fœtal vessels beyond the placenta ; but he had spoken of the continuation of the venous canals of the mother into the placenta, where, according to him, they dilate into large sinuses, upon the walls of which the placental tufts are not only ramified, but also project into the interior, carrying the walls of the sinuses before them, much in the manner described by Dr. Reid. The chief difference between the two sets of views consists therefore in this,—that Dr. Reid considers the interior of the placenta in the light of one large sac, continuous with the uterine vessels, having its cavity divided by the interlacement of the ramifications of the fœtal vessels, which are covered by the reflexions of the lining of the sac, into numerous small cells communicating with each other, whilst, according to Weber, several smaller cavities or sinuses are contained in the substance of that organ.

Those who are curious in Morphological Science will not have much difficulty in perceiving a strong analogy between the mode in which the fœtal blood is thus renewed, by being brought into relation with the maternal fluid, and that in which the blood is aerated in the mollusca. The relative positions of the placenta and the branchiæ (the structure of which has been shown to be essentially the same) in respect to the heart, rectum, &c., have a very close correspondence.—*Edinburgh Med. and Surg. Journal.*

---

*On Bleeding in Mania.* By W. A. F. Browne, M. D., Superintendent of the Dumfries Asylum.—Dr. Browne not only condemns the use of bloodletting in mania, but goes so far as to say that "in nine cases out of ten, when a professional man even of eminence is called to a case of recent mania, he orders general depletion, the liberal exhibition of the solution of tartar emetic, brisk cathartics, and cold applications to a shaven scalp. If the symptoms, and especially the violent and rapid pulse, continue or return in unabated force, the patient is perhaps bled again from the arm, or, if not, he will be cupped and leeched to a certainty. And after these energetic measures are manfully urged for days or weeks, and the tartar-emetic lustration fails in every respect, except in producing a nausea quiet, an asylum is recommended as the last resource." Dr. Browne then states that such treatment is not confined to mania: "The melancholic monomaniac and maniac, and all the species included under these general terms, are its objects or its victims. The subject is too momentous for ridicule; but ridiculous and indefensible must that system be which includes all the foregoing diseases under one category, and draws blood and drenches upon the same principle and with the same degree of discrimination as a leech."

We agree entirely with Dr. Browne in thinking the subject too momentous for ridicule, but we consider it as at the same time too momentous for exaggeration; and we cannot help fearing that in his anxiety to warn his readers against the injury arising from the indiscriminate use of the lancet in mental affections, Dr. Browne has unconsciously and unjustifiably allowed himself to bring a strong general charge against his brethren, which, if true to the extent which he alleges, would be a reproach and disgrace to them. We believe that in many cases bleeding and tartar emetic *are* used where they had been better omitted. But so far as our information and experience go, we have no hesitation in affirming that instead of nine out of ten intelligent general practitioners following the culpable course here ascribed to them, those who do so constitute only a small minority of the whole. From the strong language used by Dr. Browne, and his charge being specially extended to the intelligent and eminent among general practitioners, we are forced to the conclusion that he must have either overstated his case, or been unusually unfortunate in meeting with so many instances of mismanagement. We concur cordially with him in his objections to general bleeding and nauseating in mania, in his anxiety for the early removal of the insane to an asylum, in the general propriety of a generous diet, and we are as earnest as he can be that no cases of maltreatment should ever occur. But for that very reason we contend that he ought either greatly to have restricted his charge against his professional brethren, or *to have produced proofs to substantiate it.* We know that cases such as he describes are to be met with more frequently than is creditable to the parties concerned; but after twenty years' experience, we can honestly aver that instead of their amounting to nine cases out of ten, of those treated by well-informed and sensible practitioners, as Dr. Browne assumes, they have been few and far between compared with those treated without general depletion. And on inquiry among our medical acquaintance, we should say that instead of nine out of ten practitioners, "even of eminence," falling into the error imputed to them, the large majority would condemn it as heartily as Dr. Browne himself. Appearing, as this statement now does, *without proofs*, it is calculated to rouse in the minds of Dr. Browne's brethren an unfriendly feeling towards himself, and thereby to diminish his influence when really in advance of them, by diminishing their confidence in his accuracy and judgment.—*Ib., from Edin. Month. Jour.*

---

*On the action of certain Inorganic Compounds, when introduced directly into the Blood.* By James Blake, Esq., M. R. C. S.—The present paper is a continuation of a memoir read at the Académie des Sciences of Paris, in 1839, and entitled " Effêts de diverses substances salines, injectées dans le systême circulatoire. " (See Achives Générales de Médecine, Nov. 1839.) After some preliminary remarks, the author gives a list of the various substances of which he noted the effects, when they were severally injected into the venous or arterial systems, arranged according to the nature of these effects. The substances experimented on were the salts of magnesia, zinc, lime, strontia, baryta, lead, silver, soda, potass, and ammonia. The author carefully inquires into the phenomena, apparently arising from the direct contact of each of these substances with the animal tissues, and more particularly into the effects produced on the heart, on the muscular and nervous tissues, and on the pulmonary and systemic capillaries. The general conclusion which the author is led to draw from these researches is, that there exists a close relation between the chemical properties of the substances experimented upon and their physiological effects; his experiments tending to prove that, when introduced into the blood, substances that are isomorphous exert similar actions on the living tissues. He notices, however, two exceptions to this law; namely, the similarity of the actions exerted on the pulmonary tissue by the salts of lead and silver, although these salts are not isomorphous; and also the action on the nervous tissue of the salts of ammonia, being different from that of the salts of potass. But he remarks that the oxide of lead bears a close analogy to the oxide of silver in its relation to organic compounds; and also that the salts of ammonia, in separating themselves from those of potass by their action on the nervous tissue, yet become closely connected in this respect with the poisons derived from organic substances, with which their chemical composition is so strikingly analogous. The general fact, previously announced by the author, in his memoir read to the Academy of Sciences, at Paris, namely, that salts with the same base have analogous actions when introduced directly into the blood, may be considered a corollary of the above law.—*Athenæum.*

---

*Observations on the Blood Discs and their contents, by* John Quekett, *Student of Human and Comparative Anatomy at the Royal College of Surgeons.* (Communicated by Dr. Pereira.)—The author's observations in the paper whose title has just been given, lead him to the following conclusions:—That each red particle of human blood is a flattened circular disc, consisting of an outer membrane or envelope, with a gelatiniform fluid in its interior, which, under certain circumstances, is capable of becoming granular, and of escaping from the envelope in the form of small globules, the general number from each disc being about six or seven; and that the discs may present either a bi-convex or bi-concave surface: the latter form being by far the most common.

The author has hitherto failed in making out the existence of a central spot or nucleus, as usually described. He declines stating for the present what he has ascertained the contained globules to be, intending to lay before the society, at some future period, not only this, but also an account of the important part played by them in some of the effects of inflammation.

The author describes, at considerable length, the appearances presented by the granules, both in the act of escaping from the discs and afterwards, pointing out the confirmation furnished by his observations of the correctness of the description given by Leuwenhoeck, of the appearance presented by the globules of blood; that distinguished observer having described each disc as composed of six smaller ones.—The author's description was illustrated by a series of drawings of the discs and granules, as seen in the microscope.—*Lond. Med. Gaz.*

---

*Remarkable Case of Secondary Syphilis.*—A man, who had previously suffered from secondary syphilis, for which he had taken a strong decoction of sarsaparilla and hemlock pills, a year after this treatment, (no secondary symptoms having occurred during the interval,) was suddenly seized with strabismus, without any known cause; and it gradually reached such a pitch, that both pupils were turned towards the nose, whenever he wanted to look either at a distant or near object. As he had previously suffered from caries of the external bones of the nose, I was afraid that a similar disease might have arisen in the orbital bones, and that caries or exostosis in the orbit produced the phenomenon by pressure on the nerves of the eye. As the patient refused to go through a course of sarsaparilla again, on account of his occupation, I prescribed pills of corrosive sublimate, beginning with one-tenth of a grain a day, and at the same time I placed an antimonial plaster on the back of his neck. When about three three grains of the sublimate had been taken, the strabismus disappeared; it was succeeded by unquenchable thirst, and a total cessation of the secretion of the skin. The pulse became quick, evacuations by stool were difficult, unfrequent, and clayey; the patient lost his appetite, and the quantity of urine increased so much, that thirty ounces were usually evacuated in a night. The urine was as clear as water, and without odour, deposited no sediment, and, as appeared by chemical analysis, contained no sugar. Hence it was a case of diabetes insipidus, which had, perhaps, arisen from taking cold. Sudorifics, and milk of sulphur to keep the body open, soon removed these symptoms, and the patient, who was rather attenuated, recovered entirely, and is now quite well.—*Ibid., from Zeitschrift für die gesammte Medicin.*

---

*Complete Paralysis of the fifth pair on one side with complete abolition of sight, hearing, smelling and taste on the same side, cured by Galvanism.* By M. James, Interne at La Charité.—At the sitting of the Royal Academy of Medicine on the 21st of October, 1840, M. James presented a young man who had been affected for two years with paralyis of the fifth pair on one side, which had been cured by the aid of galvanism.

The patient was in the wards of M. Andral, and stated that after neuralgia of the side of the face corresponding to the paralysis, which lasted eight days, the paralysis supervened. On his admission there was absolute loss of sensibility of the whole of the skin receiving the three sensitive branches of the fifth pair. It was proved that the conjunctiva, the nasal and lingual mucous membrane, and that of the cavity of the tympanum on the unaffected side, had no tactile sensibility. The special sensibilities of these four organs were also completely abolished on the affected side. M. James was convinced that the base of the tongue did not perceive tastes on the affected side, and was not sensible of the contact of a body. The muscles receiving the nerves of the motor branch were also paralysed, as proved by the unequal depression of the jaw, with an evident displacement outwards of the paralysed side, and impossibility of closing the jaws on that side. All these functional disorders yielded to galvanism applied, in a considerable number of sittings, to the different points affected. The organs of sense first recovered their sensibility; the tactile sensibility returned afterwards.—*Brit. and For. Med. Rev. from Gazette Mèdecale de Paris.*

---

*Extraction of a Prune-stone, which had remained eleven days in the Air-passages.* By M. Bonnet, Surgeon to the Hôtel Dieu, at Lyons.—The symptoms in this case were convulsive cough, with *complete* remissions. The cough increased in severity and continuance; and eleven days after the accident, death appeared so imminent that the parents objected to an operation as useless. The chest was clear on percussion, but there was such an absence of the respiratory murmur as could only be attributed to some mechanical obstacle to the entrance of air to the lung. The signs of elevation and depression of the foreign body could not be recognized. The operation of tracheotomy was performed in the usual manner, but six ligatures had to be applied to vessels of the thyroid gland. The stone was removed by forceps, and complete recovery followed. The child was eleven years of age.—*Ib., from Bul. Gén. de Thérapeutique.*

---

*Fibres in the walls of the Gall-Bladder.*—M. Barth has presented to the Anatomical Society of Paris, a dilated gall-bladder, the walls of which offered very apparent fibres, interlaced, and having a great resemblance to muscular fibres.—*Ibid. from L'Expérience.*

